# Microencapsulated Comb-Like Polymeric Solid-Solid Phase Change Materials via In-Situ Polymerization

**DOI:** 10.3390/polym10020172

**Published:** 2018-02-11

**Authors:** Wei Li, Xiaoye Geng, Rui Huang, Jianping Wang, Ning Wang, Xingxiang Zhang

**Affiliations:** State Key Laboratory of Separation Membranes and Membrane Processes, Tianjin Key Laboratory of Advanced Fibers and Energy Storage, School of Material Science and Engineering, Tianjin Polytechnic University, Tianjin 300387, China; 18222858076@163.com (X.G.); ruihuang1225@gmail.com (R.H.); wangjianping@tjpu.edu.cn (J.W.); wangning620@tjpu.edu.cn (N.W.); zhangxingxiang@tjpu.edu.cn (X.Z.)

**Keywords:** comb-like polymer, phase change materials, microcapsule, permeability resistance, thermal stability

## Abstract

To enhance the thermal stability and permeability resistance, a comb-like polymer with crystallizable side chains was fabricated as solid-solid phase change materials (PCMs) inside the cores of microcapsules and nanocapsules prepared via in-situ polymerization. In this study, the effects on the surface morphology and microstructure of micro/nanocapsules caused by microencapsulating different types of core materials (i.e., *n*-hexadecane, ethyl hexadecanoate, hexadecyl acrylate and poly(hexadecyl acrylate)) were systematically studied via field emission scanning electron microscope (FE-SEM) and transmission electron microscope (TEM). The confined crystallization behavior of comb-like polymer PCMs cores was investigated via differential scanning calorimeter (DSC). Comparing with low molecular organic PCMs cores, the thermal stability of PCMs microencapsulated comb-like polymer enhanced significantly, and the permeability resistance improved obviously as well. Based on these resultant analysis, the microencapsulated comb-like polymeric PCMs with excellent thermal stability and permeability resistance showed promising foreground in the field of organic solution spun, melt processing and organic coating.

## 1. Introduction

Phase change materials (PCMs) exhibiting excellent thermal energy storage, which can absorb, store and release large amount of latent heat, play an important role in energy management following the increasing energy crisis. Encapsulation techniques (e.g., microencapsulation, nanoencapsulation and macroencapsulation) provide opportunities to prepare advanced PCMs with a greater heat transfer area, reduce reactivity with outside environment and control volume changes during the phase transition [1]. Therefore, microencapsulated PCMs are being applied widely in many fields including thermal-regulated fibers and fabrics [2,3], intelligent buildings [4,5], thermal energy storage [6,7,8,9,10,11,12,13] and so on.

Thermal stability of microencapsulated/nanoencapsulated PCMs (Micro/NanoPCMs) plays an important role in melt processing (e.g., melt-spun composite fiber). The maximum weight loss temperature of PCMs microencapsulated low molecular organic materials (e.g., *n*-alkenes, aliphatic alcohol, fatty acids and their derivatives) is often much lower than boiling point, which results in worse thermal stability of corresponding encapsulated PCMs [14]. In addition, permeability resistance of PCMs encapsulated low molecular materials PCMs is often unsatisfactory when styrene-based or acrylic-based copolymer [14] is used as shell materials.

A comb-like polymer is a special type of graft copolymer, in which macromonomer or oligomer molecular chains are attached to the polymeric backbones, and the excellent thermal energy storage of comb-like polymer in virtue of crystallizable side chains has attracted increasing attention. Shi et al. [15] fabricated a series of comb-like polymeric PCMs (denoted as poly(ethylene-graft-maleic anhydride)-*g*-alkyl alcohol) via an esterification reaction between poly(ethylene-graft-maleic anhydride) and *n*-alkyl alcohols. They found that the heat enthalpy changes from 125 to 146.2 J/g with the side chain length increasing, and the thermal energy storage materials fabricated could be used under 300 °C [16]. To enhance the rotational flexibility of the crystallizable side chain, Zhang et al. selected polyethylene glycol octadecyl ether as side chains instead of octadecyl alcohol to prepare the PCMs, which was still stable around 295 °C [17]. In addition, permeability of comb-like polymeric PCMs decreased obviously due to the restriction of polymeric backbones. Most of these comb-like polymers still exhibit physical gel state when side chains are in molten state, which probably limits potential applications.

Comparing with conventional Micro/NanoPCMs, both the thermal stability and permeability resistance of microencapsulated comb-like polymeric PCMs could be expected to enhance significantly. In this study, we polymerized hexadecyl acrylate as monomer using free radical polymerization inside the micro/nanocapsules shell to fabricate the comb-like polymer, which was subsequently encapsulated via in-situ polymerization. In addition, microencapsulated *n*-hexadecane, ethyl hexadecanoate or hexadecyl acrylate is also beneficial to thermal stability and permeability resistance of PCMs to some extent. Microstructure and morphology of micro/nanocapsules were characterized via FE-SEM and TEM, and thermal property of comb-like polymeric PCMs was also investigated via DSC. Furthermore, the influencing factors of thermal stability PCMs microencapsulated the comb-like polymer or low molecular materials were systematically compared and discussed via thermogravimetric analyzer.

## 2. Experimental

### 2.1. Materials

*N*-hexadecane, ethyl hexadecanoate and hexadecyl acrylate, used as core materials, were purchased from Aladdin Reagent Company (Shanghai, China). 2,2′-azobis(2-methylpropionitrile) (AIBN) purchased from Shanghai Jingchun Chemical Co., Ltd, Shanghai, China) was used as initiator. The prepolymer of methanol modified melamine-formaldehyde (MMF, 73% solid content, Aonisite Chemical Trade Co., Ltd., Tianjin, China) was employed as shell-forming monomer. An aqueous solution of the sodium salt of styrene-maleic anhydride copolymer solution (SMA, 19 wt %) employed as surfactant was kindly supplied by Institute of Functional Fiber of Tianjin Polytechnic University (Tianjin, China).

### 2.2. Fabrication of Micro/NanoPCMs (i.e., MicroH, MicroEH and MicroHA)

The encapsulation was carried out in a 500 mL three-neck round-bottomed flask. Firstly, 15.0 g SMA solution was dissolved in 135.0 g distilled water at 40 °C, and the pH value of resultant micelles solution was adjusted to 5.0–5.5 with 20.0 wt % citric acid solution. The oil phase employed as core material (Table 1, *n*-hexadecane, ethyl hexadecanoate and hexadecyl acrylate) was added to the aqueous micelles solution, and the mixture was emulsified mechanically with a stirring rate of 8000 rpm for 10 min to form an oil-in-water emulsion. The high shear emulsification machine (MBE-LOL-E, Shanghai Xinruidu Chemical Machinery Co., Ltd, Shanghai, China) was employed to prepare stable emulsion, and the rotor speed of the homogenizer ranged from 0 to 10,000 rpm. The emulsion was shifted to a 500 mL three-neck round-bottomed flask, and then purged with nitrogen to remove the oxygen air dissolved in the emulsion. Subsequently, the 15.0 g MMF prepolymer solution was dropped into the emulsion system at a rate of approximately 0.25 g·min^−1^ and kept being stirred at a speed of 500 rpm. After adding the prepolymer, the stirring speed was also increased to 1000 rpm, and the reaction temperature of the system was raised to 75 °C to promote the polycondensation and encapsulation of MMF prepolymer. For sample micro/nanoencapsulated hexadecyl acrylate (MicroHA), the free radical polymerization of hexadecyl acrylate droplets also started, accompanied by encapsulation. The encapsulation reaction continued for 180 min, and the system pH was adjusted to around 7 with 20 wt % sodium hydrate solution to terminate the reaction. The resultant micro/nanocapsules suspension product could be obtained, and then filtered with qualitative filter paper with pores size ranging from 30 to 50 μm approximately, and subsequently washed twice with 30 wt % ethanol solution at 50 °C to remove some unencapsulated oil core materials, self-polymer nanoparticles or emulsifier. The wet cake of micro/nanoencapsulated *n-*Hexadecane (MicroH), micro/nanoencapsulated ethyl hexadecanoate (MicroEH) and micro/nanoencapsulated hexadecyl acrylate (MicroHA) were obtained, respectively, and then dried in a ventilated oven at 50 °C.

### 2.3. Preparation of Micro/NanoPCMs MicroPHA

The encapsulation of Micro/NanoPCMs MicroPHA (Table 1, MicroPHA) was as follows: 15.0 g SMA was dissolved in 135.0 g distilled water. Subsequently, the core material (hexadecyl acrylate) and initiator (AIBN) employed as oil phase was added to the aqueous SMA solution at 40 °C. Then, the mixture was emulsified mechanically under a stirring rate of 8000 rpm for 10 min to form an oil-in-water emulsion. Subsequently, the emulsion was shifted to a 500 mL three-neck round-bottomed flask, and then purged with nitrogen to remove the oxygen air dissolved in the emulsion. The temperature of emulsion was raised to 75 °C to initiate the radical polymerization of hexadecyl acrylate. Then, the poly(hexadecyl acrylate) was synthesized to be microencapsulated after reaction for 180 min. Afterwards, the suspension system was cooled down to 40 °C, and the pH value was adjusted to 5.0–5.5. Then, 15.0 g MMF prepolymer was dropped into the emulsion at a rate of approximately 0.25 g·min^−1^ under a stirring speed of 500 rpm. The temperature of suspension was raised to 75 °C after the prepolymer was totally added, and the stirring speed was also increased to 1000 rpm at the same time. The encapsulation reaction continued for 180 min, and the system pH was adjusted to around 7 with 20 wt % sodium hydrate solution to terminate the reaction. Finally, the resultant micro/nanocapsules MicroPHA could be collected similar to those of MicroH, MicroEH and MicroHA mentioned above.

### 2.4. Extraction Experiment of Micro/NanoPCMs

The Micro/NanoPCMs were dried for 3 h in an oven at 110 °C, and 2.0 g capsules were sealed in a filter paper bag. Then, the bag was immerged in the extraction vessel containing hot alcohol of 50 °C and extracted for 24 h. The bag was then taken out and dried for 3 h in an oven at 110 °C. The symbol α was defined as the enthalpy loss of Micro/NanoPCMs according the following equation:

(1) $$\alpha =\frac{\left(\left|\Delta H_{1}\right|-\left|\Delta H_{2}\right|\right)}{\left|\Delta H_{1}\right|}\times 100\%$$

where |Δ*H*_1_| and |Δ*H*_2_| are the measured enthalpies of Micro/NanoPCMs without extraction and extracted microcapsules, respectively.

### 2.5. Micro/NanoPCMs Characterization

Both the morphology and microstructure of the Mico/NanoPCMs were observed by cold field emission scanning electronic microscope (FE-SEM, HITACHI, S4800, Tokyo, Japan) equipped with energy-dispersive X-ray. The core-shell structure of Micro/NanoPCMs could be observed by transmission electron microscopy (TEM, HITACHI, H-7650, Tokyo, Japan). The thermal properties data of dried Micro/NanoPCMs were investigated using differential scanning calorimetry (DSC, NETZSCH 200F3, Selb, Gemany) under nitrogen atmosphere, and the phase changes were characterized from −20 to 80 °C at heating or cooling rate of 10 °C·min^−1^ after elimination of the thermal history. The thermal stabilities of dried Micro/NanoPCMs were characterized by thermogravimetric analyzer (TGA, Netzsch409pc, Selb, Gemany) at a heating rate of 10 °C·min^−1^ with the temperature range of 25–800 °C.

### 2.6. Encapsulation Efficiency of Micro/NanoPCMs

The theoretical content of Micro/NanoPCMs (*C*_t_) fabricated via in-situ microencapsulation can be obtained from the Equation (2)

(2) $$C_{t}=\frac{M_{\mathrm{core}}}{\left(M_{\mathrm{core}}+M_{\mathrm{shell}}+M_{\mathrm{emulsifier}}\right)}\times 100\%$$

where *M*_core_, *M*_shell_ and *M*_emulsifier_ stand for the weight of PCMs added, pure MMF prepolymer added and pure SMA added, respectively.

The actual measured content of Micro/NanoPCMs (*C*_m_) can be estimated according to the measured enthalpy:

(3) $$C_{m}=\frac{\left|\Delta H\right|}{\left|\Delta H_{0}\right|}\times 100\%$$

where ΔH is the average of melting enthalpy and crystallizing enthalpy measured by DSC of Micro/NanoPCMs, and $\Delta H_{0}$ is the average of melting enthalpy and crystallizing enthalpy of bulk PCMs.

The Encapsulation efficiency of Micro/NanoPCMs (*E*_e_) is defined as following:

(4) $$E_{e}=\frac{C_{m}}{C_{t}}\times 100\%$$

## 3. Results and Discussion

### 3.1. Mechanism of In-Situ Encapsulation

**Figure 1:**
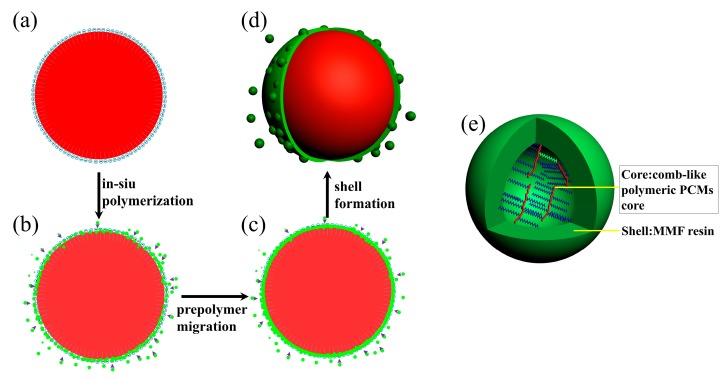
Schematic diagram of encapsulation process via in-situ polymerization: (**a**) oil core droplets, (**b**) microencapsulation process, (**c**) crosslinking process, (**d**) microcapsule; and (**e**) schematic structure of Micro/NanoPCMs containing comb-like polymeric phase change materials.

Figure 1 shows the schematic fabrication of Micro/NanoPCMs process by in-situ polymerization. Organic PCMs (i.e., *n*-hexadecane, ethyl hexadecanoate, and hexadecyl acrylate) used as oil phase were emulsified mechanically in aqueous phase to form oil-in-water emulsion. An aqueous solution of the sodium salt of styrene-maleic anhydride copolymer (SMA) used as surfactant was dissolved in aqueous solution, where the hydrophilic polar groups (–COO^–^) alternatively arranged along the backbone of copolymer. At the interface, the emulsifiers oriented their polar groups (–COO^–^) towards the aqueous phase and their non-polar region towards the oil phase of droplets, thus the surface of the oil droplets were negatively charged [18]. This orientation of SMA molecule not only resulted in a stable oil-in-water emulsion, but also provided a relatively strong electron negative field on the surface of oil droplets, as shown in Figure 1a.

When the shell-forming MMF prepolymer was dropped in continuous acidic aqueous phase, the positively charged MMF prepolymer and hydrophilic carboxylate acid ion located on SMA attracted each other by the charge effect. The electric attraction was dominant at early stage, so most of MMF prepolymer was adsorbed onto the oil particles under electrostatic attractive force and thus polycondensation occurred subsequently to form core-shell structure. Moreover, a small amount of prepolymer would form nanoparticles on the shell surface via self-polymerization, particularly when the electric attraction becomes weaker after a certain reaction period. The nanoparticles are liable to aggregate and then absorb on the surface of the microcapsules primarily by physical absorption due to their high surface energy, as illustrated in Figure 1c,d. Similarly, oil droplets containing hexadecyl acrylate and AIBN used as initiator were emulsified in continuous aqueous phase to form stable oil-in-water emulsion. While the oil droplets were being encapsulated by MMF resin shell, the monomers inside the cores started to polymerize, initiated by thermal decomposition of AIBN, thus the core-shell structure of Micro/NanoPCMs containing comb-like polymeric poly(hexadecyl acrylate) as core materials could be fabricated, as presented in Figure 1e.

### 3.2. Morphology of Micro/NanoPCMs Containing Various Phase Change Materials

**Figure 2:**
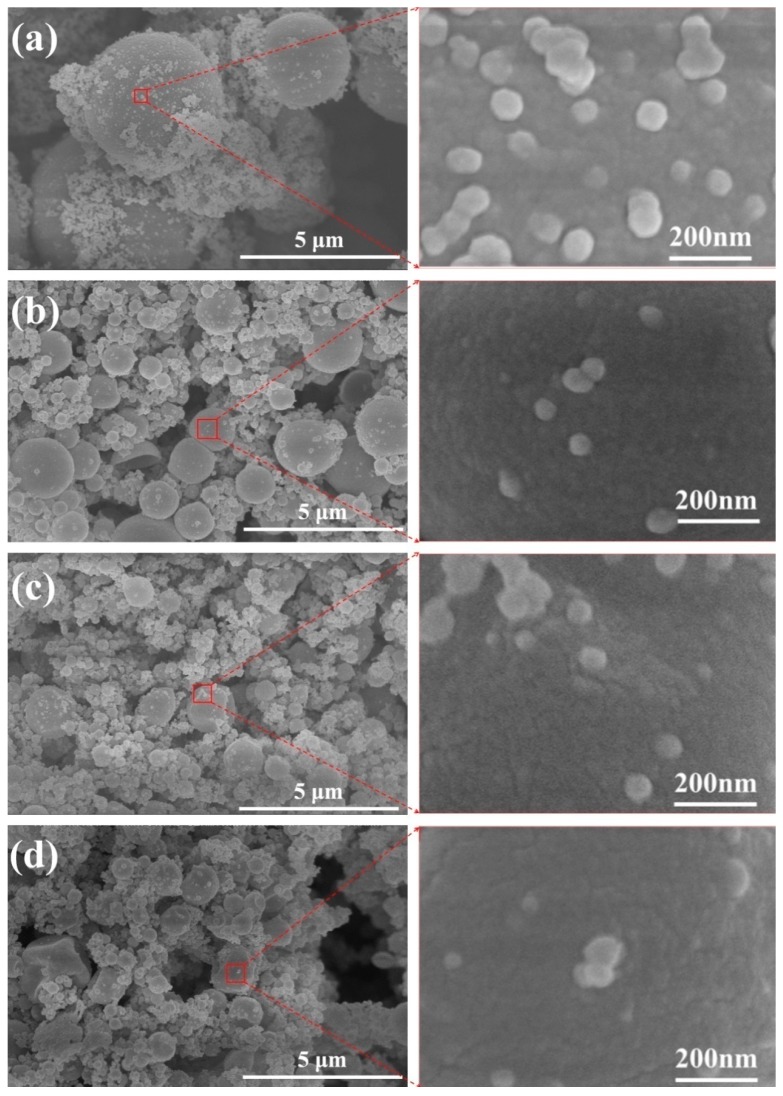
SEM micrographs of Micro/NanoPCMs containing various core materials fabricated with SMA concentration of 10 wt %: (**a**) MicroH; (**b**) MicroEH; (**c**) MicroHA; and (**d**) MicroPHA.

The effects of various PCMs core on morphology of Micro/NanoPCMs could be observed via FE-SEM, as shown in Figure 2. The MMF prepolymer could not completely cover the cores, and some MMF self-polymer nanoparticles adhered onto the shell outer surface or between capsules. Compared with the other three samples, more polymer nanoparticles ranging from 10 to 100 nm existed on the surface of Micro/NanoPCMs MicroH. However, the nanoparticles number reduced dramatically with a decrease of Micro/NanoPCMs diameter, as shown in Figure 2b–d, which might attribute to an increase of the contact probability between shell-forming prepolymer and cores which caused by the increase of core specific surface area. As discussed in “Mechanism of In-Situ Encapsulation”, in the continuous phase, the positively charged MMF prepolymers and SMA macromolecules carrying carboxylate acid ion could attract each other via charge effect, and the prepolymers added firstly would be adsorbed to the interface of oil droplets surrounded with SMA macromolecules when electric attraction was dominant at early stage. The enriched prepolymer around the oil droplets would start condensation polymerization to form MMF resin shell. However, with the electric attraction decreasing, some prepolymers would form nanoparticles ranging from 10 to 100 nm. These heterogeneous polymerized nanoparticles were mainly composed of MMF polymer and SMA micelles, and they tended to aggregate and then absorb on the surface of the microcapsules primarily by physical absorption due to their high surface energy. The diameters of Micro/NanoPCMs containing comb-like polymeric PCMs (i.e., MicroPHA) as core were in the range of 100 nm–2.5 μm, and the difference between sample MicroHA and MicroPHA was not obvious. Moreover, the capsule surface presented as sunken, which was mainly caused by the shrinkage of comb-like poly(hexadecyl acrylate) when phase transition occurred.

### 3.3. Effects of Surfactant on the Surface of Micro/NanoPCMs

**Figure 3:**
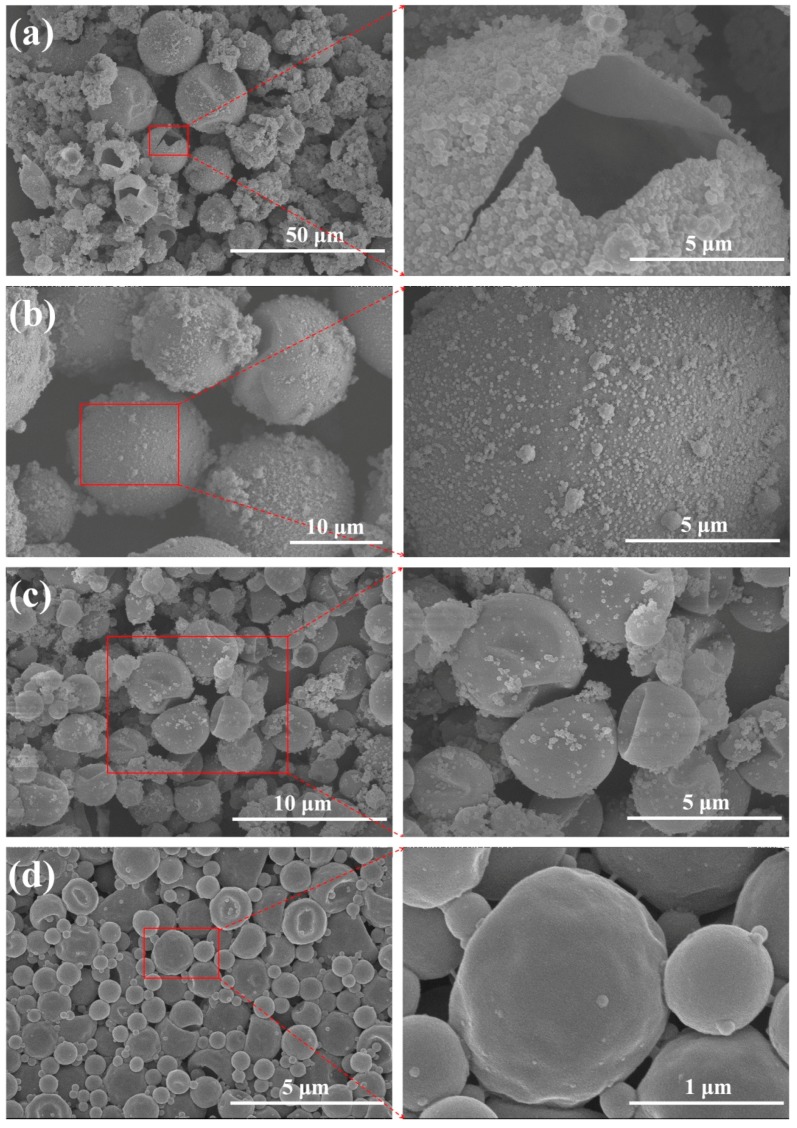
SEM micrographs of Micro/NanoPCMs containing *n-*dodecanol with various concentration of SMA: (**a**) 5.0 wt %; (**b**) 6.7 wt %; (**c**) 10.0 wt %; and (**d**) 15.0 wt %.

**Figure 4:**
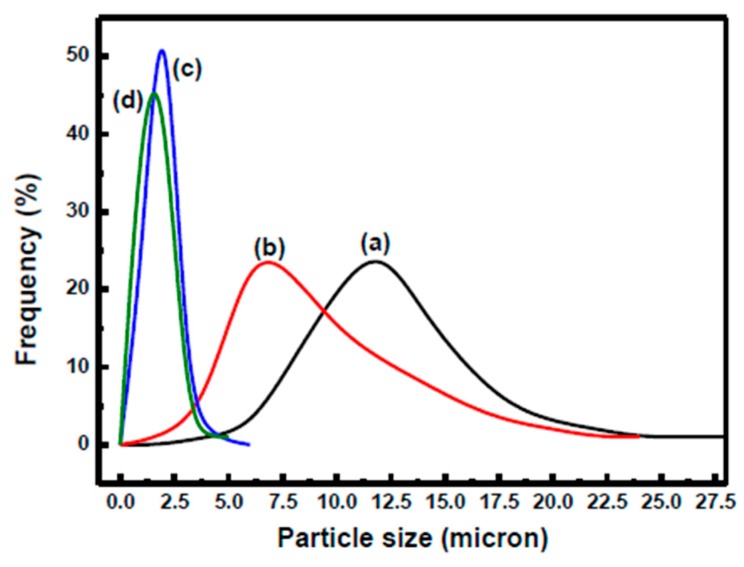
Diameter distribution of Micro/NanoPCMs fabricated with various concentration of SMA: (**a**) 5.0 wt %; (**b**) 6.7 wt %; (**c**) 10.0 wt %; and (**d**) 15.0 wt %.

To further investigate the influence of the parameters involved in the nanoparticles attaching to the surface of Micro/NanoPCMs, a series of capsules with various diameters were fabricated, as presented in Figure 3. The organic PCM *n*-dodecanol was selected as core material, and stable emulsion containing nano-scale droplets could be easily obtained via high shear emulsification due to its unique chemical structure. With the concentration of surfactants SMA increasing from 5 to 15 wt %, the average diameter regularly decreased from approximately 11.7 to 1.5 μm and size distribution became narrower as well, as shown in Figure 4. The internal structure of the microcapsules fractured was hollow and had a single internal cavity, as shown in Figure 3a, indicating the forming of core-shell structure. The outer shell, which was different from the smooth and compact inner layer of shell, was rough and absorbed some nanoparticles mainly resulted from self-polymerization of MMF prepolymer. It is noteworthy that fewer and fewer nanoparticles were found on the surface as the diameter decreased, and the surface turned compact and smooth gradually. The reason might be that the reaction sites increased during encapsulation process. The particle size of droplets in emulsions decreased with an increase of the amount of emulsifier, which resulted in higher specific surface area and more encapsulation reaction sites. Therefore, less MMF prepolymer covered on the surface of emulsions drops might be the reason that the self-polymerized nanoparticles reduced.

### 3.4. Core-Shell Microstructure of Micro/NanoPCMs Containing Comb-Like Polymeric PCMs

**Figure 5:**
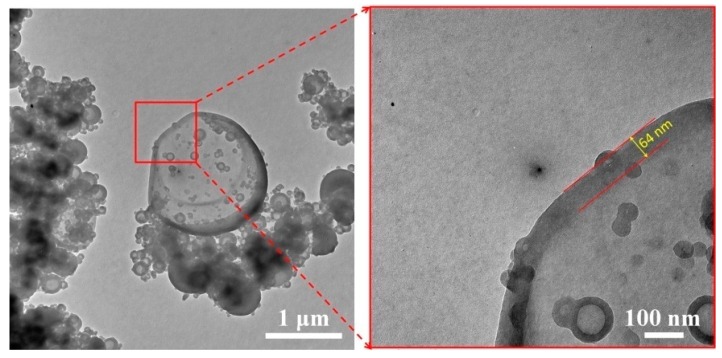
TEM micrographs of Micro/NanoPCMs microencapsulated comb-like polymeric PCMs.

The internal microstructure of Micro/NanoPCMs could be observed via TEM, as shown in Figure 5. The particle size and distribution of Micro/NanoPCMs were measured in accordance with the SEM micrographs in Figure 2c. More importantly, obvious core-shell structure of Micro/NanoPCMs could be observed and demonstrated, and the shell thickness was close to 64 nm. Some core-shell structured nanocapsules with diameter less than 100 nm could also be found. These heterogeneous polymerized nanoparticles ranging from 10 to 100 nm were located on the capsule owing to physicochemical force, and were solid particles mainly composed of MMF polymer and SMA micelles.

### 3.5. Thermal Stability of Various PCMs and Micro/NanoPCMs

**Figure 6:**
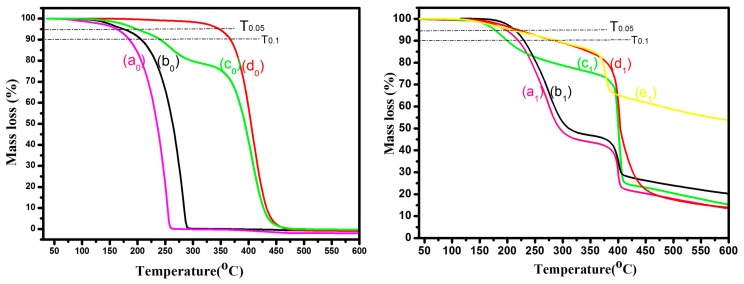
TGA curves comparison of various PCMs and Micro/NanoPCMs: ($a_{0}$) *n*-hexadecane; ($b_{0}$) ethyl hexadecanoate; ($c_{0}$) hexadecyl acrylate; ($d_{0}$) poly(hexadecyl acrylate); ($a_{1}$) MicroH; ($b_{1}$) MicroEH, ($c_{1}$) MicroHA; ($d_{1}$) MicroPHA; and ($e_{1}$) MMF shell.

The thermal stability of Micro/NanoPCMs, which plays an important role in thermal energy storage application (e.g., melt processing), was characterized by the weight loss mechanism via dynamic thermogravimetry analysis [19]. In Figure 6$a_{0},b_{0},d_{0}$, the *n*-hexadecane, ethyl hexadecanoate and poly(hexadecyl acrylate) had a single step weight loss process, probably resulting from evaporation or decomposition of pure PCMs materials. The weight loss end temperatures were around 261.6, 291.6 and 466.8 °C, respectively, thus demonstrating the outstanding thermal stability of comb-like polymeric solid-solid PCMs poly(hexadecyl acrylate). A two-step weight loss process appear in the TGA curve of hexadecyl acrylate (Figure 6$c_{0}$). The first step is probably attributable to the evaporation of hexadecyl acrylate monomer. With the temperature increasing during TGA measurement, self-polymerization of hexadecyl acrylate might occur due to the high temperature even without initiator, which is why the second step temperature was so close to the decomposition point of poly(hexadecyl acrylate) presented in Figure 6$d_{0}$. The *T*_0.05_ and *T*_0.1_ temperature of various PCMs and Micro/NanoPCMs on the TGA curves are listed in Table 2.

The *T*_0.05_ and *T*_0.1_ temperature of poly(hexadecyl acrylate) were 346.8 and 366.8 °C, respectively, which was much higher than traditional organic PCMs including *n*-hexadecane and ethyl hexadecanoate. The reason might be that different molecular weight and chemical structure resulted in various bonding force among PCMs molecules or macromolecules. Compared with pure PCMs, the thermal stability of microencapsulated *n*-hexadecane and microencapsulated ethyl hexadecanoate (Figure 6$a_{1}$,$b_{1}$) were enhanced by 23.4–38.4 °C, which is attributable to the protection of MMF resin shell. The *T*_0.05_ and *T*_0.1_ temperature of poly(hexadecyl acrylate) decreased to certain extent after being encapsulated by MMF shell, which is probably attributable to the small molecules such H_2_O, CH_3_OH or CH_2_O released from further polycondensation or decomposition of MMF resin shell, and the insufficient polymerization of hexadecyl acrylate inside the capsule cores. As presented in Figure 6$a_{1}$–$d_{1}$, the second step weight loss process of these Micro/NanoPCMs were similar, which might be related with the structural decomposition of MMF resin shell.

The *T*_0.05_ and *T*_0.1_ of microencapsulated poly(hexadecanoate acrylate) increased 56.4 and 120.5 °C compared to the traditional alkane PCMs (e.g., hexadecane), respectively, which indicated that this kind of microencapsulated comb-like polymeric PCMs could resist higher temperature during melt processing.

### 3.6. Phase Change Properties and Permeability of Various Micro/NanoPCMs

**Figure 7:**
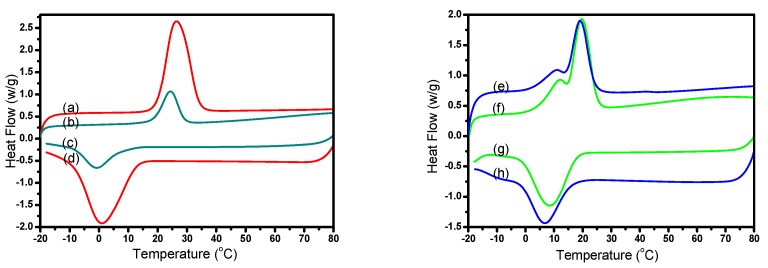
DSC curves comparison of various Micro/NanoPCMs: (a,d) microencapsulated *n*-hexadecane; (b,c) microencapsulated *n*-hexadecane after extraction by alcohol; (f,g) microencapsulated poly(hexadecyl acrylate); and (e,h) microencapsulated poly(hexadecyl acrylate) after extraction by alcohol.

The thermal properties data of Micro/NanoPCMs measured via DSC are summarized in Table 3 and the phase change behaviors were characterized from −20 to 80 °C, as shown in Figure 7. The in-situ polymerization is always considered as an optimal microencapsulation strategy with high encapsulation efficiency, and the encapsulation efficiency of microencapsulated *n*-hexadecane could be obtained as high as 89.8% according the Equations (2)–(4). Due to the confined crystallization of PCMs inside the capsule shells [20] and thermal conductivity of polymer shells [21,22], the supercooling degree of microencapsulated poly(hexadecyl acrylate) was measured as close to 11.2 °C, which is 14.2 °C lower than that of microencapsulated *n*-hexadecane. The endothermic enthalpy and exothermic enthalpy of Micro/NanoPCMs microencapsulated poly(hexadecyl acrylate) were 78.9 and 61.0 J/g, respectively, and the low energy storage capacity relating to imperfect crystal structure could attribute to confined crystallization side chains along the polymeric chains. The sealness of Micro/NanoPCMs, as a key factor for blending process and energy storage, has an important effect on the permeability of MMF resin shell. Fan et al. determined the permeability of microcapsules by weight loss of extracted microcapsules, which is calculated by enthalpy method [23]. It is worth noting that the enthalpy loss of microencapsulated poly(hexadecyl acrylate) and microencapsulated *n*-hexadecane after extraction in ethanol is calculated as 17.1 % and 72.7% according to Equation (1). Such relatively low enthalpy loss rate demonstrated the micro microencapsulated poly(hexadecyl acrylate) possessing excellent permeability. Compared with *n*-hexadecane, these crystallizable side chains attached to polymeric backbones of poly(hexadecyl acrylate) probably became more difficult to permeate through the MMF resin shell. Furthermore, it can be concluded that the microencapsulated comb-like polymeric PCMs with good tightness could be suitable for versatile application field such as organic coating and organic solution spun.

## 4. Conclusions

In this study, microencapsulated comb-like polymeric poly(hexadecyl acrylate) with crystallizable side chains was prepared via in-situ polymerization. The effects of types of core materials (i.e., *n*-hexadecane, ethyl hexadecanoate, and poly(hexadecyl acrylate)) on the surface morphology, microstructure and phase change properties were systematically discussed. The thermal stability temperature *T*_0.05_ and *T*_0.1_ of microencapsulated poly(hexadecanoate acrylate) increased 56.4 and 120.5 °C compared to the traditional alkane PCMs (e.g., hexadecane), respectively, which indicated that this kind of microencapsulated comb-like polymeric PCMs could resist higher temperature during melt processing. The supercooling degree of microencapsulated poly(hexadecyl acrylate) was 11.2 °C, which was much lower than the 25.4 °C of microencapsulated *n*-hexadecane. The enthalpy loss of microencapsulated poly(hexadecyl acrylate) after extraction in ethanol was 17.1% and the relatively low enthalpy loss rate demonstrated the microencapsulated poly(hexadecyl acrylate) possessing excellent permeability. However, the energy storage density of Micro/NanoPCMs with poly(hexadecanoate acrylate) core was approximately 70.0 J/g, probably caused by confined crystallization, which means that its phase change property might need to be enhanced further for energy storage or thermos-regulated field.

## Figures and Tables

**Table 1:** Component content of oil phase core.

	Sample	MicroH ^a^	MicroEH ^b^	MicroHA ^c^	MicroPHA ^d^	MicroD ^e^
Oil core						
*n-*Hexadecane (g)		15	-	-	-	
ethyl hexadecanoate (g)		-	15	-	-	
hexadecyl acrylate (g)		-	-	15	15	
AIBN ^f^ (g)		-	-	0.3	0.3	
*n-*dodecanol						15

^a^ micro/nanoencapsulated *n-*Hexadecane; ^b^ micro/nanoencapsulated ethyl hexadecanoate; ^c^ micro/nanoencapsulated hexadecyl acrylate; ^d^ micro/nanoencapsulated polymerized hexadecyl acrylate; ^e^ micro/nanoencapsulated *n-*dodecanol; ^f^ 2,2′-azobisisobutyronitrile.

**Table 2:** Thermal stability comparison of various PCMs and Micro/NanoPCMs.

Sample	H	MicroH	EH	MicroEH	HA	MicroHA	PHA	MicroPHA	MMF
*T*_0.05_	161.6	196.0	174.1	212.5	196.7	176.6	346.8	218.0	205.4
*T*_0.1_	184.1	218.5	206.6	230.0	239.2	199.1	366.8	286.6	289.4

*T*_0.05_, the temperature of 5% weight loss; *T*_0.1_, the temperature at 10% weight loss.

**Table 3:** Phase change performance comparison of Micro/NanoPCMs between before extraction and after extraction.

Sample	*T*_mp_^a^ (°C)	△*H*_m_^b^ (J/g)	*T*_cp_^c^ (°C)	△*H*_c_^d^ (J/g)	△*H* ^e^ (J/g)	*T*_sc_^f^ (°C)	*Α* ^g^ (%)
MicroH ^h^	26.4	110.4	1.0	124.8	117.6	25.4	
*MicroH ^i^	24.4	28.8	−0.7	35.4	32.1	25.1	72.7
MicroPHA ^j^	19.7	78.9	8.5	61.0	70.0	11.2	
*MicroPHA ^k^	19.2	60.5	6.8	55.5	58.0	12.4	17.1

^a^ Peak temperature on DSC heating curve; ^b^ Enthalpy on DSC heating curve; ^c^ Peak temperature on DSC cooling curve; ^d^ Enthalpy on DSC cooling curve; ^e^ Average enthalpy of |Δ*H*_m_| and |Δ*H*_c_|; ^f^ Supercooling degree = *T*_mp_−*T*_cp_; ^g^ Enthalpy loss of Micro/NanoPCMs after extraction; ^h^ microencapsulated *n*-hexadecane; ^I^ microencapsulated *n*-hexadecane after extraction; ^j^ microencapsulated poly(hexadecyl acrylate); ^k^ microencapsulated poly(hexadecyl acrylate) after extraction.
